# The Action of Liquor Potassæ on the Urine in Health

**Published:** 1853-11

**Authors:** 


*The action of Liquor Potassæ on the Urine in health.* By E. A. Parkes, M. D., &c., &c.

Dr. Parkes thus recapitulates the results of his long and laborious paper upon this subject:

If this remedy be taken soon after meals, its action is that of an antacid. It combines with hydrochloric or with lactic acid, and then, doubtless, passes into the circulation. What appreciable effect it now produces is not indicated in the tables above given, but it does not increase either the water, solids, or sulphuric acid of the urine. If the liquor potassæ be taken into an empty stomach, it passes unneutralized into the circulation, and probably through the veins; in so doing it must produce an effect on the walls of the capillaries and small veins, but the extent of this cannot be known. As much as ʒij. have been taken with only 4oz. of water, without causing epigastric pain or uneasiness, (although it produced considerable temporary scalding of the mouth and throat,) and without apparently producing any local effects in the stomach. In, usually, from thirty to ninety minutes after its entrance into the circulation, an increased flow of slightly acid urine occurs, which contains the whole of the potash, organic matter differing considerably from that of ordinary urine, and a relatively large proportion of sulphuric acid; the phosphoric acid and the chlorine are less changed. Perhaps an organic acid, (not uric, and probably not hippuric,) is also present. The explanation of these facts is, that an albuminous compound, either in the blood itself, or in the textures, has become oxidized; its sulphur, under the form of sulphuric acid, has united with potash, and, with possibly the changed protein-compound, is poured out from the kidneys. This oxidizing effect of the liquor potassæ is, no doubt, assisted by exercise, and by copious draughts of water; but in the above experiments, exercise and fluid were abstained from, in order not to complicate the results. The amount of albumen or fibrine destroyed by one drachm of liquor potassæ, cannot be considerable, but if the potash were continued in large quantities, oxidation could probably be pushed to any amount. The nitrate and acetate of potash did not in a *healthy system* have the same effects.

After the increased flow of urine, the quantity passed per hour falls slightly below the standard. It appears to resume its ordinary composition, but its exact condition at this period, has not been determined. Some observations on urine in disease would lead me to infer that the uric acid will be found to be increased. Such were the effects of liquor potassæ on the urine. The effect produced on other excretions was not obvious. The skin and the intestines appeared quite unaffected, and as all the potash was found in the urine, the reason of this is easily understood. In most of the experiments there were no subjective symptoms of any kind. On two occasions, there was rather sharp frontal headache, languor, depression, slight lumbar pain, and aching of the legs, after the large flow of urine. On the night of the 15th, when the flow of urine, which was proceeding at the rate of ℥iss. per hour, was augmented in two and a half hours by ℥xiv., and no fluid was supplied to the system, the pulse became perceptibly small, (almost thready,) and slow: it remained equal and regular—there was no thirst, no shiverings, and no nausea; the skin was dry and warm. In six hours the pulse had quite regained its force and frequency, and the other symptoms had disappeared without any fluid having been taken.

After the experiments were concluded, the general health did not appear impaired; it was, if anything, better than usual. The effect of liquor potassæ on the diseased system, is a much more difficult problem. The chemical conditions are not the same, and the effects of the potash are necessarily influenced by them. I will not now enter into this subject, but observe that it is necessary, when its oxidizing effects are desired, to give the potash eight or ten hours after food, to drink moderate quantities of water, and, if possible, to use exercise. The potash should be given pure, or with large doses of iodide of potassium, but unmixed with sugar. I may so far anticipate what will be hereafter said on this point, by stating that, administered in this way, it exerts a powerful effect on the exudations of inflammations, but appears less useful in the early stages, when an antagonistic force seems to be in action. It remains to be seen whether the varying excretion of sulphuric acid, which is unaccounted for by diet and exercise, is occasioned by greater or less alkalinity of the blood producing variations in the amount of oxidation of the albuminous compounds.—*Dublin Medical Press*, from *Med. Chir. Review.*

